# Aged interleukin-10^tm1Cgn^ chronically inflamed mice have substantially reduced fat mass, metabolic rate, and adipokines

**DOI:** 10.1371/journal.pone.0186811

**Published:** 2017-12-21

**Authors:** Reyhan M Westbrook, Huan Le Yang, Jackie M Langdon, Cindy N Roy, Jin A Kim, Parichoy P Choudhury, Qian-Li Xue, Andrea di Francesco, Rafa de Cabo, Jeremy Walston

**Affiliations:** 1 Division of Geriatric Medicine and Gerontology, Johns Hopkins University School of Medicine, Baltimore, MD, United States of America; 2 Department of Biostatistics, Johns Hopkins Bloomberg School of Public Health, Baltimore, MD, United States of America; 3 Experimental Gerontology Section, Translational Gerontology Branch, National Institute on Aging, National Institutes of Health, Baltimore, MD, United States of America; Universidade do Estado do Rio de Janeiro, BRAZIL

## Abstract

Interleukin 10^tm1Cgn^ (IL 10^tm^) mice have been utilized as a model of chronic inflammation and declining health span because of their propensity to develop chronic activation in NFkB pathways, skeletal muscle and cardiac changes, and mitochondrial dysfunction. We hypothesized that older IL 10^tm^ frail mice would have alterations similar to frail, older humans in measured parameters of glucose metabolism, oxygen consumption (VO_2_), respiratory quotient (RQ), spontaneous locomotor activity, body composition and plasma adipokine levels. To test this hypothesis, we investigated these metabolic parameters in cohorts of 3, 10, and 20 month old IL 10^tm^ female mice and age and gender matched C57Bl/6 mice. Insulin sensitivity, glucose homeostasis, locomotor activity and RQ were not significantly altered between the two strains of mice. Interestingly, old IL 10^tm^ mice had significantly decreased VO_2_ when normalized by lean mass, but not when normalized by fat mass or the lean/fat mass ratio. NMR based body composition analysis and dissection weights show that fat mass is decreased with age in IL 10^tm^ mice compared to controls. Further, plasma adiponectin and leptin were also decreased in IL 10^tm^.These findings suggest that frailty observed in this mouse model of chronic inflammation may in part be driven by alterations in fat mass, hormone secretion and energy metabolism.

## Introduction

Chronic low-grade inflammation is a common feature of aging and frailty. Frailty is a geriatric syndrome characterized by multisystem dysregulation yielding increased vulnerability to stressors, decreased physiological reserves, and altered metabolism, which leads to a limited capacity to maintain homeostasis [1,2]. Understanding the connection between chronic inflammation and the biological changes that underlie late life vulnerability, frailty, and health span decline in older adults is crucial to the development of targeted novel interventions to reduce propensity to chronic disease states and to adverse health outcomes in vulnerable individuals. The development of animal models that approximate age and inflammation induced health span declines in humans is necessary to facilitate etiologic and treatment-focused frailty research.

A mouse homozygous for a targeted deletion in the interleukin 10 (IL-10) gene B6.129P2-*IL 10*^*tm1Cgn*^/J (IL 10^tm^) is increasingly utilized as a mouse model for health span, chronic illness, frailty, and low-grade inflammation research [3–11]. Interleukin 10 (IL-10) is a cytokine with pleiotropic effects in immunoregulation and inflammation [12]. Importantly, IL-10 can block NF-κB activity and is involved in regulation of the JAK-STAT signaling pathway. Thus IL-10 can inhibit the synthesis of a number of inflammatory cytokines. The lack of the anti-inflammatory cytokine IL-10 in this mouse leads to increased mean serum IGF-1, and increased expression of NF-κB-induced inflammatory mediators including: IL 1-β, TNF-α, IFN-γ, IL-6 and chemokine ligand-1 [8,13,14]. With chronic systemic activation in inflammatory pathways early in life, these mice undergo accelerated changes consistent with late life decline including muscle and bone weakness, altered skeletal muscle gene profile, IGF-1 changes, cardiac dysfunction, lower mitochondrial energy production, and alterations in the renin-angiotensin system pathway [3–11].

Aging, chronic inflammation, and frailty are associated with impaired insulin sensitivity and glucose homeostasis, decreased metabolic rate and locomotor activity, as well as altered respiratory quotient (RQ) and body composition in humans [15]. Although IL 10^tm^ mice lack changes in these phenotypic parameters early in life [16–18], little is known about the impact of age and chronic inflammation on body composition, insulin resistance and other metabolic changes, and how these factors contribute to previously observed late life decline and premature mortality in these mice [4,6]. The identification of important age and inflammation driven metabolic and body composition differences between the IL 10^tm^ and age and gender matched C57Bl6 mice may provide crucial physiologic evidence which may facilitate molecular investigation into the processes that connect chronic inflammation to frailty and late life decline in older adults. With this goal in mind, experiments were designed to answer the question: how does the low grade inflammatory state of the IL 10tm mice affect metabolic and body composition parameters across the lifespan?

## Methods

### Animals

Cross-sectional comparisons of young (5 month old), middle aged (12 month old), and old, (22 month old) female IL-10 deficient B6.129P2-IL 10tm1Cgn/J (IL 10^tm^) mice and age- and sex-matched C57Bl/6 (control) mice were undertaken. IL 10^tm^ mice were homozygous for the *IL 10tm1Cgn* targeted mutation and were fully backcrossed on B6 background [8]. All female IL 10^tm^ mice were bred in the Johns Hopkins Bayview Vivarium, where they were kept under barrier conditions per prior management recommendations (Baltimore, MD). Female C57Bl/6 controls were purchased from the Jackson Laboratory (Bar Harbor, ME, USA). For experimental testing, all mice were shipped to the National Institute on Aging Extramural Branch in Baltimore, Maryland, where they were housed and where all *in vivo* experiments were conducted. All experimental procedures performed in this study were approved by the Johns Hopkins University animal care and use committee (protocol number: MO12M341).

Mice were housed in 75 in^2^, autoclave sterilized, high-temperature polycarbonate shoebox cages in ventilated racks (Allentown Inc., Allentown, NJ, USA) containing autoclaved corncob bedding (Harlan Teklad, Indianapolis, IN, USA), autoclaved mouse chow 2018SX (Harlan, Teklad), and reverse osmosis-filtered hyperchlorinated water dispensed through an in-cage automatic watering system (Edstrom Industries, Waterford, WI, USA). Rooms were maintained at 22 ± 3.6°C on a 14-h light/10-h dark cycle with automated monitoring by Siemens Building Technologies, Inc. (Zurich, Switzerland). Cages were changed every 2 weeks in laminar airflow change stations (The Baker Co., Sanford, ME, USA) with surface cleaning and disinfection with MB-10 disinfectant (Quip Laboratories Inc., Wilmington, DE, USA). All caging was sanitized by automatic cage washing systems and autoclaved prior to use

### GTT and ITT

For the glucose tolerance test (GTT), mice (n = 10 per age & genotype group) were fasted overnight (~12 hours) and on the subsequent morning, injected i.p. with 2g glucose/kg body weight. Blood samples (amount to fill capillary in standard glucometer; approximately 0.002ml per sample) was collected from the tail at baseline, 15, 30, 60 and 120 min after injection. For collection of the first sample, an approximately 3–4 mm knick near the tip of the tail was cut with a sharp razor blade and the tail gently massaged. For collection of consecutive blood sample, the scab was removed with clean tissue. For the insuIin tolerance test (ITT), fully fed animals were injected i.p. with 0.75 IU of insulin per kg body weight and blood samples were collected as described for the glucose tolerance test.

### Body composition

The amounts of body fluid, fat, and lean body mass were determined in whole live mice (n = 10 per age & genotype group). The assessment of total fat, lean, and fluid mass for each individual animal was acquired by nuclear magnetic resonance (NMR) using the Minispec LF90 (Bruker Optics, Billerica, MA). These measurements are then compared to total body mass to acquire the relative percentage of fat, lean and fluid mass. At the time of sacrifice, mice (n = 83 controls & n = 82 IL 10^tm^) were anesthetized with isoflurane and all tissues and organs were harvested and weighed by trained lab personnel. Whole blood was collected with BD Microtainer tube with Dipotassium EDTA (Becton, Dickinson and Company, Franklin Lakes, NJ) and spun at 1000 g for 13 minutes then plasma was snap frozen in liquid nitrogen.

### Indirect calorimetry

Mouse metabolic rate was assessed by indirect calorimetry in open-circuit oxymax chambers using the Comprehensive Lab Animal Monitoring System (CLAMS; Columbus Instruments, Columbus, OH). Mice (n = 10 per age & genotype group) were housed singly with *ad libitum* access to food and water and maintained at 20–22°C under a 12:12-h light-dark cycle (light period 0600–1800). Sample air was passed through an oxygen sensor for determination of oxygen content. Oxygen consumption was determined by measuring oxygen concentration in air entering the chamber compared with air leaving the chamber. The sensor was calibrated against a standard gas mix containing defined quantities of oxygen, carbon dioxide and nitrogen. Constant airflow (0.5 L/min) was drawn through the chamber and monitored by a mass sensitive flow meter. The concentrations of oxygen and carbon dioxide were monitored at the inlet and outlet of the sealed chambers to calculate oxygen consumption. Measurement in each chamber was recorded for 30 s at 30-min intervals for a total of 48 h. Each animal was assessed for 3 days in the chambers while only the second and third dark:light cycles were kept and plotted. Locomotor activity (both horizontal and vertical) was concomitantly monitored with system-integrated infrared beams 0.5 inches apart on the horizontal plane, providing a high resolution grid covering the XY-planes and the software provides counts of beam breaks by the mouse in 30-s epochs. Ambulatory activity satisfies locomotor movement as the subject must move from one beam break to the next consecutive beams showing movement along the sensors. This is relative to the subject wandering about the chamber; locomotor movement.

### ELISA

Total adiponectin was measured (n = 37 IL 10^tm^, n = 33 controls) using the ALPCO (Mouse) Total Adiponectin ELISA (ALPCO Diagnostics, Salem, NH) using 10 μl samples in duplicate per the manufacturer’s instructions. The sensitivity of this assay was 0.032 ng/ml.

Plasma leptin was measured (n = 38 IL 10^tm^, n = 33 controls) using the ALPCO Leptin ELISA (ALPCO Diagnostics, Salem, NH) using 30 μl samples in duplicate per the manufacturer’s instructions. The sensitivity of this assay was 10 pg/ml.

Plasma IL- 6 was measured (n = 37 IL 10^tm^, n = 33 controls) using the mouse IL-6 High Sensitivity ELISA (eBioscience, Inc., Sandiego, CA) using 50 μl samples in duplicate per the manufacturer’s instructions. The sensitivity of this assay was 0.21 pg/ml.

Serum A1AT levels were measured (n = 21 IL 10^tm^, n = 20 controls) with ELISA kits using 2 μl samples in duplicate according to the instructions provided by Immunology Consultants Laboratory (Newberg, OR). The sensitivity of this assay was 1.492 ng/ml.

### Metabolic panel

The metabolic panel was performed (n = 33 IL 10^tm^, n = 28 controls) using the Vet Ace® Clinical Chemistry system (Alfa Wasserman Diagnostic Technologies LLC, West Caldwell, NJ). This automated bench top random access analyzer provides quantitative determination of constituents in blood including: cholesterol (total assay precision <2.3%CV, reportable range is 2–600 mg/dL), HDL (total assay precision <4.5%CV, reportable range is 2–125 mg/dL), triglycerides (total assay precision <2.0%CV, reportable range is 6–1000 mg/dL), creatine kinase (total assay precision <4.2%CV, reportable range is 3–1800 U/L), glucose (total assay precision <1.7%CV, reportable range is 1–750 mg/Dl), total protein(total assay precision <2.0%CV, reportable range is 0–14 g/dL), and albumin (total assay precision <2.8% CV, reportable range is 0.2–5.6 g/dL).

### Complete blood count (CBC)

Whole blood samples (n = 6 per age & genotype group) were analyzed for CBC by the Hemavet 950 (Drew Scientific, Waterbury, CT).

### Circulating parameters

Data generated from plasma was measured in samples collected at the time of death. Due to the time lapse between in vivo testing and sacrifice, the animals were not in all cases the same age as they were when they were initially tested. In some cases, plasma from additional mice, which were of the appropriate age, sex, and genotype, were added to the plasma sample set (circulating parameters) in order to increase the sample size. Therefore, in order to include all of the data points from the circulating parameters, we chose to display this data as a scatter plot with age as the x-axis rather than bar graphs. In the younger animals, which are of smaller size and therefore more difficult to extract substantial volumes of plasma, there are fewer data points because we simply didn’t have enough plasma to measure all parameters.

### Immunohistochemistry

Frozen pieces of visceral fat were placed in formalin for 48 hours, then embedded in paraffin and sectioned. CD11b macrophage/ monocyte specific antibody (abcam; ab133357) was used for immunohistochemistry using the protocol included in the supplement (S1 File). For measurements, we randomly sampled five 418.24 μm X 555.3 μm areas per animal from old IL 10^tm^ and control mice (n = 6 per group). Adipocyte size and CD11b stain measurements calculated using ImageJ.

### Statistical methods

Statistical analyses were performed using GraphPad Prism 6 and R statistical environment. For indirect calorimetric data we utilized a permutation based approach to test for the global difference between the average oxygen consumption between two groups using the L^2^ area under the squared difference curve as the test statistic. We compute this test statistic using a Riemann sum approximation. Under the null hypothesis of no difference in oxygen consumption between groups, the genotype labels are exchangeable. Hence the empirical distribution of the test statistic over permuted datasets approximates the distribution of the global difference between the groups under the null hypothesis. We compute the p-value by the proportion of permutations in which the estimated test statistic exceeds the observed value of the same in the given dataset and compared with two-way repeated measures ANOVA.

Student t-test was used to analyze activity body composition and complete blood count data. ITT and GTT data were analyzed using the two-way repeated measures ANOVA. Adipokine measurements and the metabolic panel were analyzed using linear regression analysis. Using body composition data from dissection, we sought to explore the relationship between body composition and circulating hormones in the same animals. To do this we included the masses of collected tissues (log transformed as necessary) as explanatory variables in a multiple linear regression model.

## Results

### Body weight & body composition

Prior to indirect calorimetry, body weight and NMR measured body composition were recorded (Table 1). Body weight was higher at baseline in 5 month old IL 10^tm^ (p = 0.007) mice and lower in 12 month old IL 10^tm^ mice (p = 0.028) compared to control mice. No significant difference was observed between the mouse strains in the 22 month old cohort. While increased percent fat mass was increased in IL 10^tm^ mice in the 5 month old group compared to controls (p = 0.001), average percent fat mass was markedly reduced in 22 month old IL 10^tm^ mice compared to controls (p = 0.01). Interestingly, percent lean mass was increased in 22 month old IL 10^tm^ mice compared to controls (p = 0.04), likely due to decreased percentage fat mass. While IL 10^tm^ mice had increased percent fluid mass at 12 months (p = 0.04) compared to controls, this was reversed in the old aged cohort with decreased percent fluid mass observed in the IL 10^tm^ (p = 0.001).

**Table 1:** NMR-based body composition.

	IL 10^tm^ 5 months Mean (SD)	C57Bl/6 5 months Mean (SD)	p-value	IL 10^tm^- 12 months Mean (SD)	C57Bl/6 12 months Mean (SD)	p-value	IL 10^tm^ 22 months Mean (SD)	C57Bl/6 22 months Mean (SD)	p-value
**Body Weight**	24.15 (0.84)	21.55 (0.20)	**0.0074**	26.24 (0.84)	30.36 (1.44)	**0.0283**	29.68 (0.87)	28.23 (1.06)	0.3056
**Lean Mass (%)**	60.42 (1.05)	61.11 (0.73)	0.1071	60.72 (2.04)	59.66 (1.28)	0.1863	62.72 (4.13)	59.44 (1.94)	**0.0352**
**Fat Mass (%)**	15.87 (1.66)	13.64 (0.81)	**0.0014**	18.24 (3.43)	21.36 (3.74)	0.0765	13.9 (6.25)	20.15 (1.17)	**0.0143**
**Fluid Mass (%)**	6.72 (0.29)	6.95 (0.25)	0.1133	6.95 (0.25)	6.67 (0.30)	**0.0444**	6.51 (0.26)	6.88 (0.14)	**0.0008**

NMR-based body composition from young, middle and old aged mice (n = 10 per group) at the time of indirect calorimetry testing. These values were used to normalize oxygen consumption measurements. SD = standard deviation.

### Indirect calorimetry

**Fig 1:**
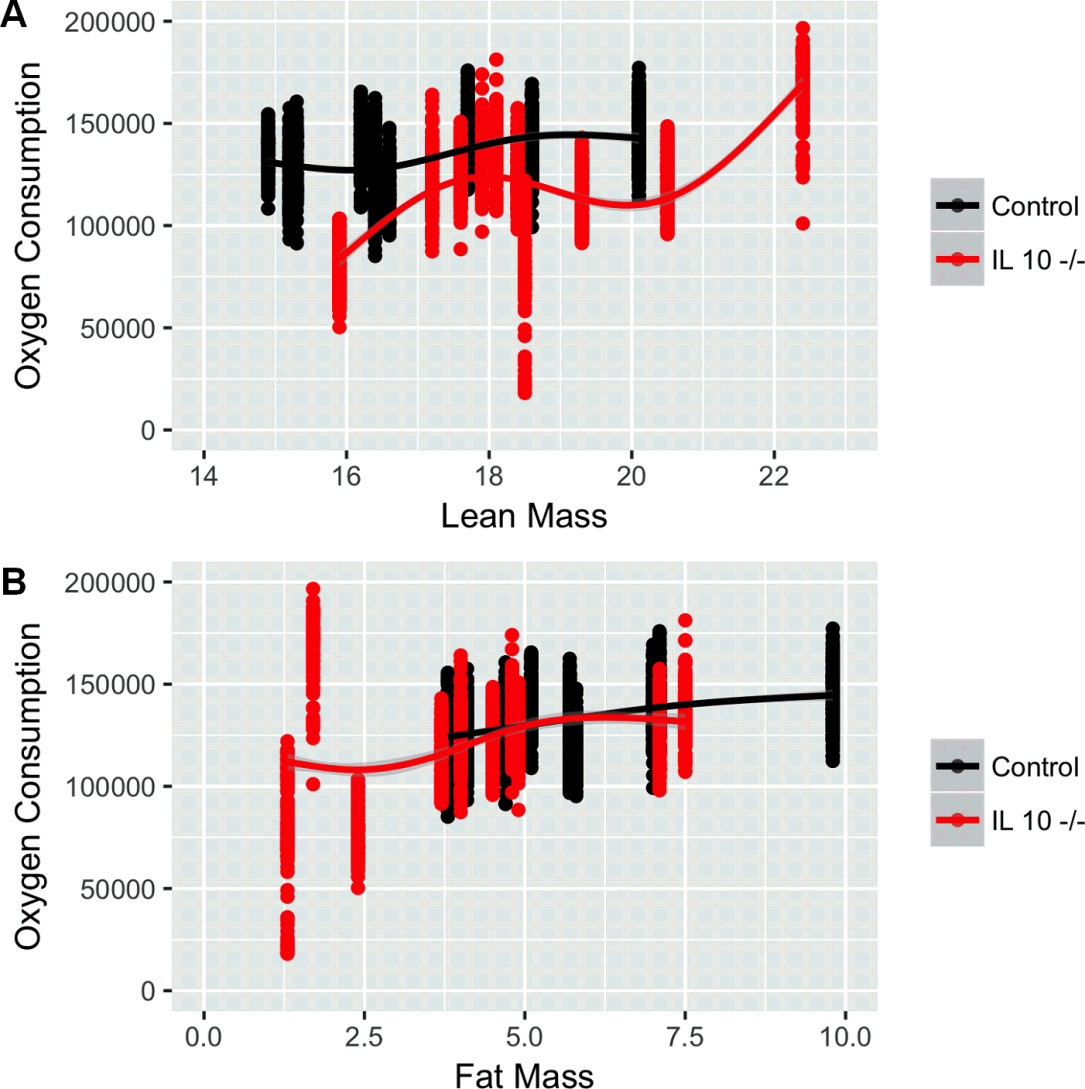
Oxygen consumption normalized by lean mass (top) and fat mass (bottom) in IL 10^tm^ (n = 10, 22 months old). (p<0.0001: Fig 1A & Fig 1B respectively). Each animal’s range of 48 hour VO_2_ is depicted by a single vertical group of points with a line connecting the means of each animal across each genotype.

Indirect calorimetry was utilized to measure oxygen consumption (VO_2_) rate as well as its rate of carbon dioxide production (VCO_2_). Interestingly, while no significant differences in VO_2_ were seen in the 5 month old (Panels A and B in S1 Fig) and 10 month old cohorts (Panels C and D in S1 Fig), VO_2_ is significantly decreased in IL 10^tm^ when normalized by lean mass (p<0.0001: Fig 1A). Normalization of VO_2_ using fat mass (Fig 1B) causes the genotype effect to disappear. These results were consistent with total mass (p = 0.019: Panel A in S2 Fig) and lean mass (p = 0.004: Panel B in S2 Fig) normalizations of this data plotted across time on the x-axis, showing decreased VO_2_ in old IL 10^tm^ mice.

RQ is the ratio comparing the volume of carbon dioxide an animal produces over time (VCO_2_) to VO_2_ (RQ = VCO_2_/V O_2_). This dimensionless ratio yields an estimate of the type of fuel substrate being oxidized for an animal’s energy needs at a given time and is often used to monitor an animal’s transition between carbohydrate (RQ = 1.0) and lipid (RQ = 0.7) oxidation. RQ was decreased (two-way repeated measures ANOVA, p<0.0001) at old age but not significantly altered in any other age groups (S3 Fig), indicating alterations in the use of metabolic fuel substrate in old IL 10^tm^mice.

**Fig 2:**
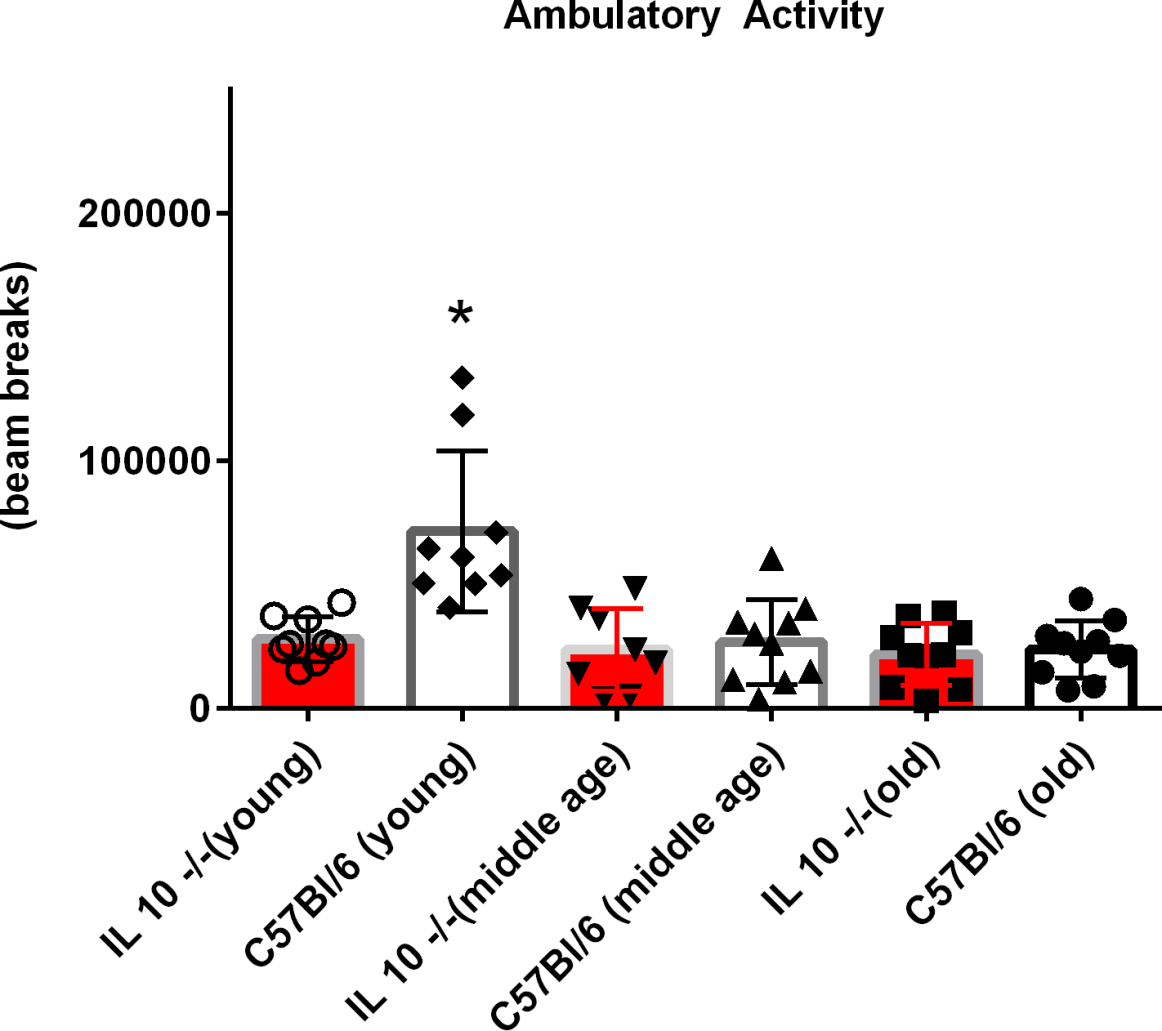
Ambulatory activity measured during indirect calorimetry using an infrared beam grid & sensors. This measurement quantifies motor movement while the subject wanders about the chamber.

Ambulatory activity was significantly decreased in young IL 10^tm^ mice compared to controls (p = 0.02), but was not significantly altered in the 12 or 22 month old cohorts (Fig 2). Z axis activity (rearing) was also decreased only in young IL 10^tm^ mice (S4 Fig). Neither food consumption nor change in body weight during testing significantly differed between genotypes at any age group (S10 Fig).

### Insulin & glucose handling in IL 10^tm^ mice

One of the most broadly accepted ways to determine glucose tolerance and insulin sensitivity is to conduct a Glucose Tolerance Test (GTT) and/or an Insulin Tolerance Test (ITT). Glucose tolerance and insulin tolerance tests involve measurements of glucose in serially collected blood samples after administration of a single dose of glucose or insulin to observe the time dependent change in glucose from the exogenous glucose or insulin challenge.

**Fig 3:**
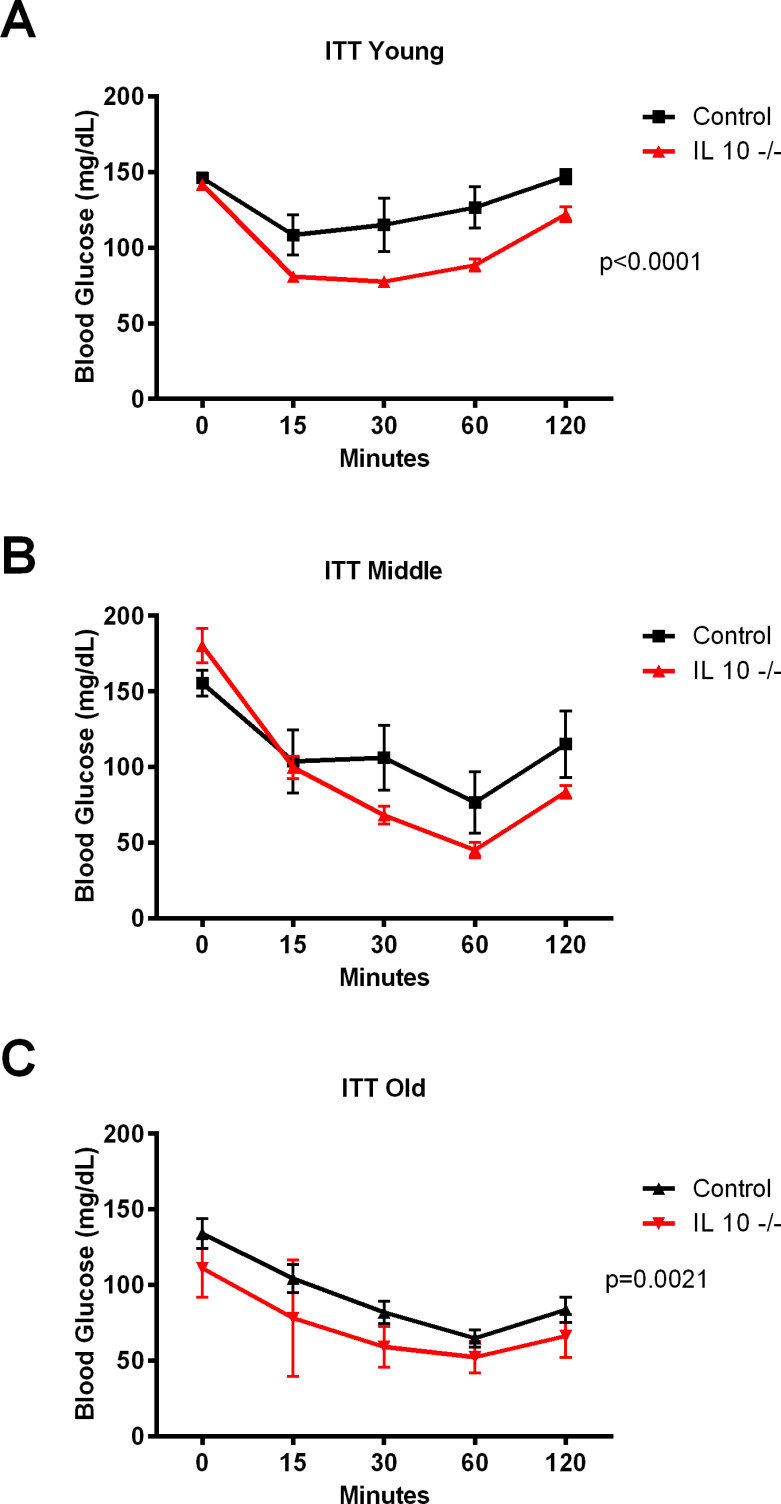
**Insulin tolerance test** in young (Fig 3A), middle aged (Fig 3B) and old (Fig 3C) mice.

The repeated measures ANOVA test of the ITT data indicated significant genotype effect in 5 month old (p<0.0001: Fig 3A), and 22 month old (p = 0.0021: Fig 3C) IL 10^tm^ mice compared to controls. Along with a similar trend in the 12 month old group, these ITT results indicate increased insulin sensitivity in IL 10^tm^ mice. Both intraperitoneal GTT’s (Panels A-C in S5 Fig), and oral GTT’s (Panels A-C in S6 Fig) showed no differences in any age cohort.

### Dissection data

Upon sacrifice each mouse was carefully dissected and tissues weighed for an in depth assessment of body composition (Table 2). Significant differences between strains at young age include increased body weight (p = 0.002) and percent visceral fat (p = 0.024), and decreased percent tibialis anterior (p = 0.0005), quadriceps (p<0.0001) and gastrocnemius (p<0.0001) in IL 10^tm^ mice. At middle age, body weight (p<0.0001), percent visceral fat (p = 0.0010) and percent subcutaneous fat (p = 0.0005) were increased in IL 10^tm^ mice while percent kidney (p<0.0001), percent tibialis anterior (p<0.0001), percent quadriceps (p<0.0001), percent gastrocnemius (p<0.0001), percent extensor digitorum longus (p = 0.008), percent triceps (p<0.0001), and percent soleus (p = 0.0098) were decreased. Changes in the old group included decreased body weight (p = 0.014), percent brown fat (p = 0.006), percent visceral fat (p = 0.016), percent perinephric fat (p = 0.009), percent subcutaneous fat (p = 0.017) and percent extensor digitorum longus (p = 0.024) as well as increased percent liver (p = 0.003) in old IL 10^tm^ mice. Interestingly, the body weight of IL 10^tm^ mice is altered relative to controls in different ways across lifespan. However, a comprehensive figure which plots the age and body weights of all mice used in the study shows that early in life, L10^tm^ have increased body weight relative to controls, however this reverses late in life with IL 10^tm^ mice weighing less than controls (S7 Fig).

**Table 2:** Tissue weights at the time of sacrifice.

	IL 10^tm^ 6 months Mean ± SEM n = 16	C57Bl/6 6 months Mean ± SEM n = 16	p-value	IL 10^tm^ 12 months Mean ± SEM n = 21	C57Bl/6 12 months Mean ± SEM n = 14	p-value	IL 10^tm^ 23 months Mean ± SEM n = 19	C57Bl/6 23 months Mean ± SEM n = 23	p-value
**Body Weight**	24.71 ± 0.51	22.60 ± 0.35	**0.0019**	33.62 ± 1.19	25.75 ± 0.65	**< 0.0001**	27.80 ± 0.70	32.14 ± 1.4	**0.0135**
**Heart** **(% of BW)**	0.577 ± 0.025	0.572 ± 0.022	0.9032	0.432 ± 0.017	0.488 ± 0.025	0.078	0.590 ± 0.028	0.539 ± 0.027	0.2010
**Liver** **(%of BW)**	5.107 ± 0.126	4.879 ± 0.146	0.2464	4.903 ± 0.153	4.873 ± 0.158	0.8938	6.511 ± 0.375	5.125 ± 0.261	**0.0034**
**Kidney** **(% of BW)**	1.531 ± 0.152	01.747 ± 0.144	0.3112	1.054 ± 0.036	1.289 ± 0.023	**< 0.0001**	1.507 ± 0.079	1.500 ± 0.072	0.9502
**Brown Fat** **(% of BW)**	0.335 ± 0.069	0.266 ± 0.021	0.3479	0.357 ± 0.017	0.363 ± 0.023	0.8129	0.303 ± 0.021	0.407 ± 0.027	**0.0056**
**Visceral Fat** **(% of BW)**	2.286 ± 0.271	1.564 ± 0.139	**0.0244**	3.446 ± 0.390	1.606 ± 0.211	**0.0010**	1.450 ± 0.353	2.723 ± 0.341	**0.0159**
**Perinephric Fat** **(% of BW)**	0.477 ± 0.021	0.394 ± 0.075	0.3076	0.779 ± 0.088	0.562 ± 0.144	0.1949	0.480 ± 0.120	1.210 ± 0.200	**0.0094**
**Subcutaneous Fat** **(% of BW)**	0.919 ± 0.047	0.782 ± 0.050	0.0565	2.074 ± 0.176	1.174 ± 0.110	**0.0005**	1.117 ± 0.296	2.134 ± 0.270	**0.0166**
**Tibialis Anterior** **(% of BW)**	0.353 ± 0.011	0.425 ± 0.014	**0.0005**	0.282 ± 0.008	0.355 ± 0.009	**< 0.0001**	0.244 ± 0.012	0.259 ± 0.010	0.3195
**Quadriceps** **(% of BW)**	1.271 ± 0.034	1.456 ± 0.015	**< 0.0001**	1.117 ± 0.027	1.389 ± 0.028	**< 0.0001**	0.860 ± 0.050	0.943 ± 0.037	0.1841
**Gastroc-** **nemius** **(% of BW)**	0.978 ± 0.026	1.123 ± 0.017	**<0.0001**	0.801 ± 0.021	0.963 ± 0.022	**< 0.0001**	0.725 ± 0.036	0.771 ± 0.028	0.3210
**Extensor Digitorum Longus** **(% of BW)**	0.092 ± 0.003	0.102 ± 0.007	0.2264	0.062 ± 0.003	0.073 ± 0.002	**0.0082**	0.047 ± 0.004	0.060 ± 0.003	**0.0242**
**Triceps** **(% of BW)**	0.771 ± 0.033	0.862 ± 0.034	0.0667	0.702 ± 0.018	0.837 ± 0.025	**< 0.0001**	0.530 ± 0.035	0.598 ± 0.029	0.9097
**Soleus** **(% of BW)**	0.068 ± 0.005	0.074 ± 0.005	0.2263	0.052 ± 0.003	0.064 ± 0.002	**0.0098**	0.044 ± 0.004	0.046 ± 0.003	0.6175

Weights of tissues dissected at time of sacrifice. SEM = Standard error of the mean

### Leptin, adiponectin, IL-6 and metabolic measures

**Fig 4:**
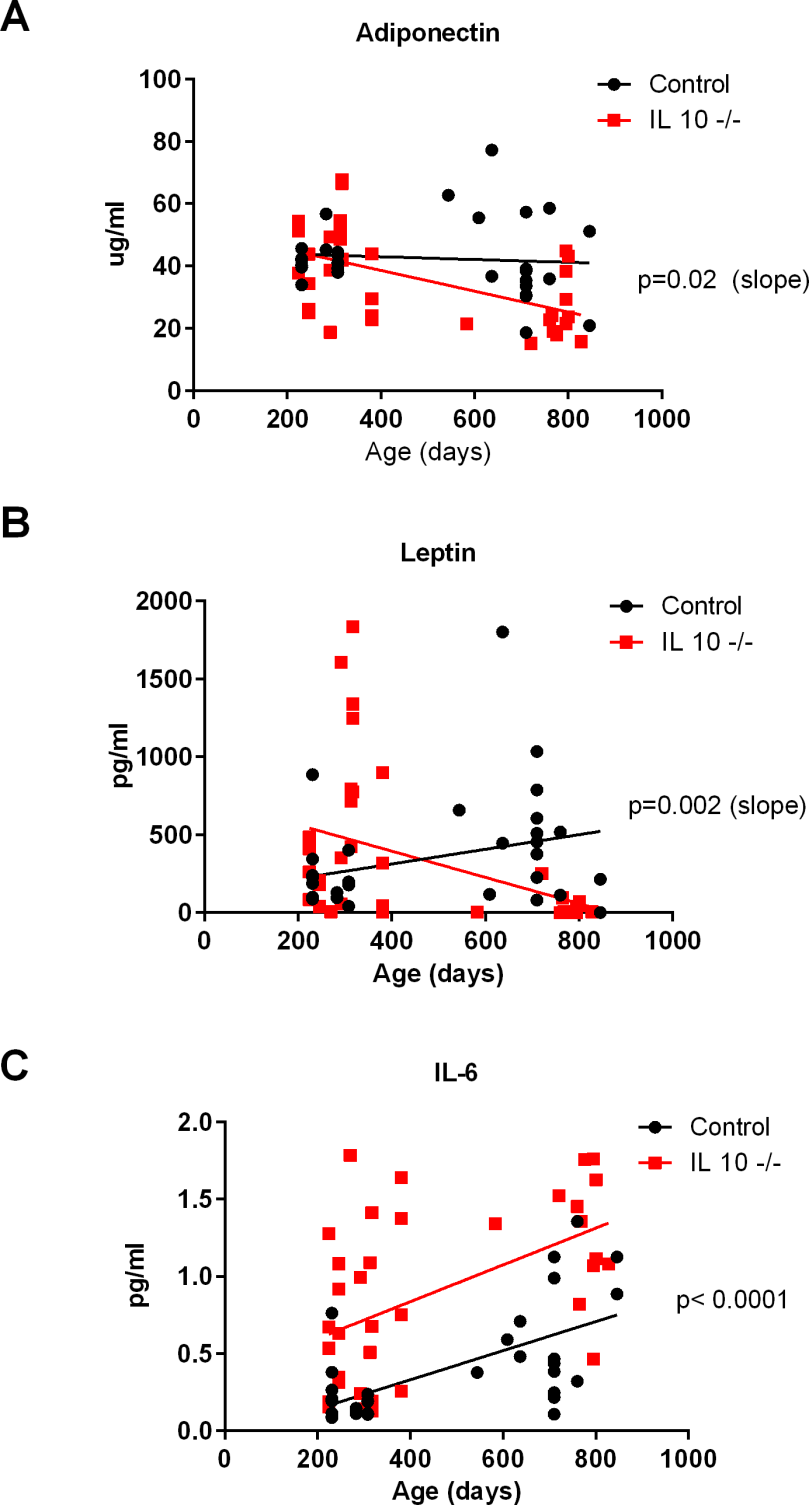
Plasma adipokine measurements across age groups at time of sacrifice. Adiponectin (Fig 4A), leptin (Fig 4B) and IL-6 (Fig 4C).

Adiponectin significantly decreased with age (linear regression analysis, p = 0.02: Fig 4A) in IL 10^tm^ mice. When we entered the masses of the different dissected tissues into a multiple regression model, the only significant predictor was percent kidney mass (T = 2.438, p = 0.03). Leptin was also significantly decreased with age in IL 10^tm^ mice compared to controls (linear regression analysis, p = 0.002: Fig 4B). When we entered the masses of the different dissected tissues into a multiple regression model, the significant predictors for leptin were total subcutaneous fat mass (T = 2.276, p = 0.037), percent subcutaneous fat mass (T = 2.91, p = 0.0102), soleus mass (log transformed, T = 4.726, p = 0.00023), perinephric fat (log transformed, T = 3.635, p = 0.002), visceral fat (log transformed, T = 2.531, p = 0.022), and age at the time of sacrifice (T = -2.23, p = 0.04). IL-6 is increased (linear regression analysis, p = 0.002) throughout life in IL 10^tm^mice (Fig 4C) and the only significant predictor was genotype (T = 3.69, p = 0.002).

### Metabolic panel

**Fig 5:**
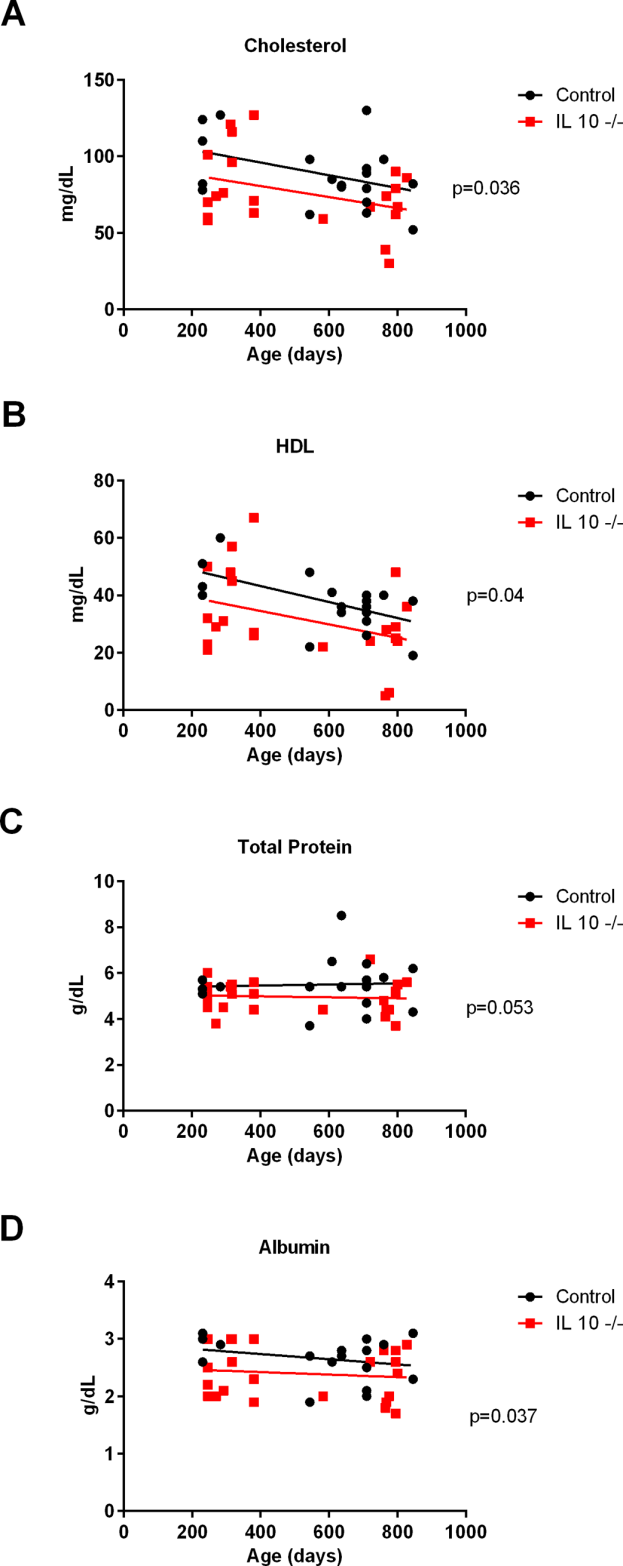
Metabolic panel. Plasma measurements of cholesterol (Fig 5A), High-density lipoprotein (Fig 5B), total protein (Fig 5C), albumin (Fig 5D).

IL 10^tm^ mice differed from controls in a number of parameters measured in plasma including decreased total (p = 0.036: Fig 5A) and HDL cholesterol (p = 0.04: Fig 5B), decreased albumin (p = 0.037: Fig 5D) and a trend for decreased total protein (p = 0.053: Fig 5C). Additionally, IL 10^tm^ mice had decreased creatine kinase (p = 0.018: Panel B in S8 Fig) and a suggestive trend for decreased blood glucose (p = 0.055: Panel A in S8 Fig).

### Alpha-1-antitrypsin (A1AT)

**Fig 6:**
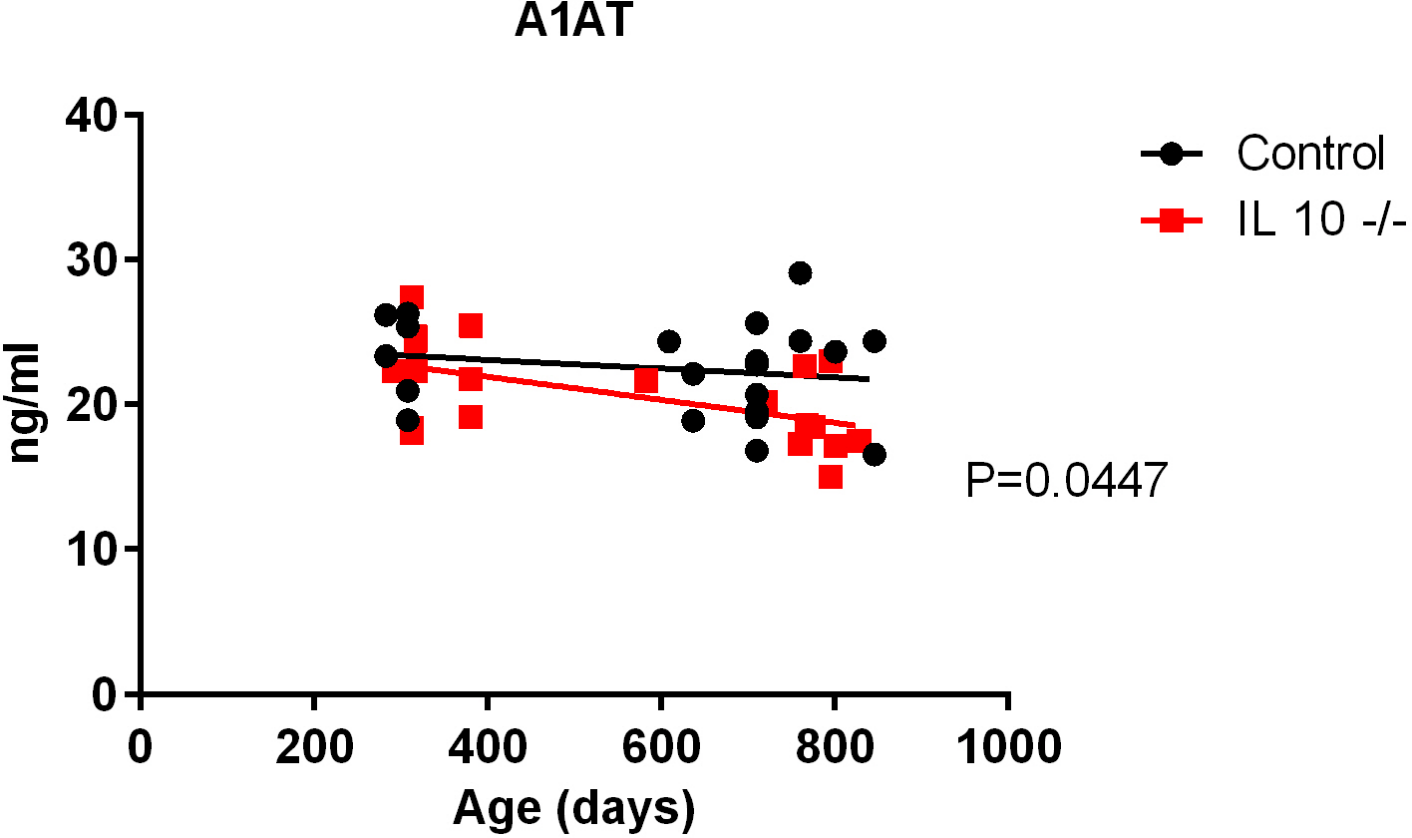
Plasma A1AT levels in IL 10^tm^ and control mice.

Alpha-1-antitrypsin (A1AT) is a 52-kDa circulating serine protease inhibitor that exhibits both tissue-protective and anti-inflammatory properties as well as immunomodulatory activities independent of protease inhibition [19–21]. Old IL 10^tm^ mice had significantly lower plasma A1AT compared to controls (linear regression, p = 0.0447: Fig 6).

### Complete blood count

There were several differences in blood cell composition as measured by the complete blood count (Table 3, S1 Table). IL 10^tm^ mice showed differences at all ages with the highest number of changes present at old age. The percentage of neutrophils was strikingly increased only in the oldest IL 10^tm^ mice with a concomitant decrease in the percentage of lymphocytes and monocytes. Interestingly, mean cell volume and mean cell hemoglobin were both significantly decreased in IL 10^tm^ mice at every time point measured. Other differences in IL 10^tm^ mice include increased red blood cells in young mice, increased platelets at middle age, and decreased mean cell hemoglobin concentration in old aged mice.

**Table 3:** Complete blood count.

	IL 10^tm^ 5 months Mean ± SEM n = 6	C57Bl/6 5 months Mean ± SEM n = 6	p-value	IL 10^tm^ 12 months Mean ± SEM n = 7	C57Bl/6 12 months Mean ± SEM n = 7	p-value	IL 10^tm^ 22 months Mean ± SEM n = 6	C57Bl/6 22 months Mean ± SEM n = 6	p-value
**Neutrophil (% of WBC)**	10.84 ± 0.89	10.57 ± 1.02	0.8491	16.51 ± 2.25	17.68 ± 3.41	0.7802	45.35 ± 5.37	20.05 ± 3.40	**0.0040**
**Lymphocyte (% of WBC)**	82.40 ± 0.94	82.59 ± 1.42	0.9147	74.58 ± 2.81	74.03 ± 4.11	0.9145	47.69 ± 5.30	69.33 ± 4.12	**0.0122**
**Monocyte (% of WBC)**	6.46 ± 0.43	6.06 ± 0.54	0.5778	6.92 ± 0.62	6.34 ± 0.37	0.4364	5.70 ± 0.75	9.04 ± 0.95	**0.0243**
**Red Blood Cell/ Erythrocyte**	10.06 ± 0.068	9.54 ± 0.19	**0.0286**	9.90 ± 0.26	9.59 ± 0.21	0.3542	8.77 ± 0.48	8.55 ± 0.23	0.6899
**Mean Cell Volume**	45.33 ± 0.40	47.75 ± 0.79	**0.0217**	44.86 ± 0.38	46.16 ± 0.44	**0.0445**	43.72 ± 0.61	46.26 ± 0.63	**0.0199**
**Mean Cell Hemoglobin**	13.27 ± 0.11	14.30 ± 0.25	**0.0036**	13.59 ± 0.12	14.23 ± 0.087	**0.0010**	13.00 ± 0.14	14.72 ± 0.11	**< 0.0001**
**Mean Cell Hemoglobin Concentration**	29.28 ± 0.28	29.93 ± 0.19	0.0808	30.27 ± 0.25	30.87 ± 0.33	0.1754	29.72 ± 0.32	31.86 ± 0.51	**0.0072**
**Platelets**	996.7 ± 29.94	877.7 ± 21.02	0.0087	1270 ± 43.03	997.7 ± 27.01	**0.0002**	1008 ± 97.21	959.8 ± 84.11	0.7163

Hematologic features of IL 10^tm^ and control mice. SEM = standard error of the means

### CD11b staining in visceral fat

**Fig 7:**
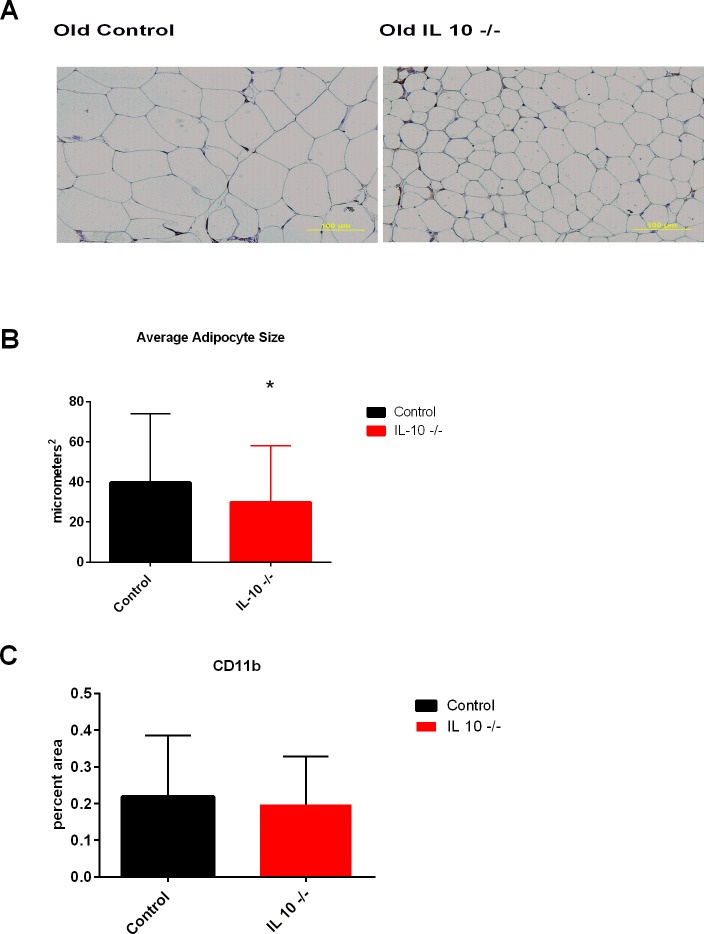
Adipocyte micrograph (Fig 7A), adipocyte size quantification (Fig 7B) and CD11b staining (Fig 7C) in visceral fat.

Old IL 10^tm^ mice had significantly smaller adipocytes (p<0.0001: Fig 7A & 7B), however there were no significant differences in the amount of CD11b positive cells in the visceral fat (Fig 7C). Additionally, histograms of adipocyte size show a shift towards smaller adipocytes in the old IL 10^tm^ mice (S9 Fig).

## Discussion

In order to better understand changes in metabolism and body composition driven by aging and chronic inflammation, and relate them to late life decline in older adults, we undertook a comprehensive characterization of female IL 10^tm^ mice at young, middle age, and old age and compared them to age and gender matched C57Bl6 background control strain mice. Key findings include a dramatic age related reduction in white adipose tissue and a concomitant decrease in levels of leptin and adiponectin secreted from fat in the IL 10^tm^ compared to control mice. Further, the oldest IL 10^tm^ mice had substantially less fat mass than control mice in the visceral, subcutaneous and perinephric adipose depots. These findings were supported by NMR body composition measurements showing a decreased percentage of body weight as fat in IL 10^tm^ mice. The loss of adipose tissue is age dependent and young mice actually had increased adipose tissue compared to controls both in our hands as well as other studies investigating adipose tissue in these mice [22].

In addition to decreased in fat mass in the old IL 10^tm^ mouse, the adipokines leptin and adiponectin were both dramatically reduced with age. Adipokines are cytokines released from adipose tissue into circulation and are involved in many metabolic processes including energy balance, immunity and inflammation [23]. Consistent with fat mass observations, leptin was higher early in life and decreased at old age in the IL 10^tm^ mice. Adiponectin level was similar to controls early in life before dropping at old age. These findings suggest that chronic inflammation in the aged IL 10^tm^ mouse may impact the function of adipocytes as well. The reduction in the levels of adipokines in the IL 10^tm^ mouse may have significant implications for metabolic function in these mice. While leptin has potent effects regulating food intake, leptin also stimulates fatty acid oxidation by up-regulating peroxisome proliferator-activated receptor γ-coactivator-1α (PPAR-alpha), and utilizing triglyceride stores within white adipocytes and liver [24]. Additionally, leptin regulates fatty acid oxidation by activating AMPK in skeletal muscle, and preventing the accumulation of lipid metabolites associated with lipotoxocity [25].

Adiponectin is also a potent regulator of energy metabolism. Adiponectin receptor activation increases AMPK and PPAR-alpha ligand activities, as well as fatty-acid oxidation and glucose uptake [26]. Adiponectin also exerts anti-inflammatory effects through its ability to suppress the nuclear factor-kappa B (NF-κB)-dependent synthesis of tumor-necrosis factor (TNF) and interferon-gamma (IFN-γ), [27] and through the induction of apoptosis of monocytes [28]. Hence the age related loss of these adipokines likely facilitates a pleiotropic effect on energy metabolism and chronic inflammation which in turn negatively impacts the healthspan of the older IL 10^tm^ mouse compared to the background control.

We also found that the oldest IL 10^tm^ mice had substantially less A1AT levels than control mice, paralleled by reduction in leptin and adiponectin levels. Beyond the impacts listed above, leptin has been shown to promote the expression of SerpinA1, the gene coding for the Ser protease inhibitor a1-antitrypsin (A1AT), [20] and the imbalance between A1AT and its natural target neutrophil elastase has been associated with reduced levels HMW adiponectin, increased leukocyte infiltration and inflammation. Although one could speculate that this decline in A1AT expression may contribute to increased fibrotic changes noted in cardiac and other tissue in older IL 10^tm^ mice [10], the metabolic consequences of this are unknown and warrant further investigation.

Resting metabolic rate is known to decrease with age in humans, and this decline is modulated by frailty status [15] Further, studies have shown decreased resting metabolic rate with decreased fat mass in elderly patients [29]. These findings are in agreement with our findings in the IL 10^tm^ mouse of decreased VO_2,_ with the percentage of fat mass being the component of body composition which most strongly correlates with this decrease. Interestingly, in some cases of frailty associated with low adiposity such as with HIV, RQ is decreased [30] as we see in this study in the IL 10^tm^ mouse.

We expected to identify a relationship between chronic inflammation and glucose intolerance in the older IL 10^tm^ mice as has been observed earlier in frail, older adults [31–33]. However, the lack of impairment in glucose or insulin handling as measured by GTT or ITT respectively between the mouse strains seen at young age [22, 34], persisted into old age. This was unexpected due to the known connection between insulin resistance and inflammation [35].

Finally, other measures yielded results that were consistent with human health span decline and frailty. IL 10^tm^ mice had decreased relative muscle mass in several muscles at different ages across the lifespan. IL 10^tm^ mice also had decreased HDL, albumin, creatine kinase, and hemoglobin. These metabolic deficiencies are known to correlate with frailty status and functional decline in human patients [36–39].

Our findings are unique in that they for the first time demonstrate declines in fat and adipokines related to chronic inflammation in this mouse model, which may help to explain other metabolic differences and a generalized lack of resiliency observed in this mouse with increasing age [4]. Strengths of the study include the use of an increasingly well characterized mouse model of chronic inflammation and careful methodological measurement [3–11]. Potential critiques of this study include the use of genetic manipulation to stimulate chronic inflammation, and the fact that absence of IL-10 could be playing an important role in this phenotype rather than the activation of chronic inflammatory pathways. Using other non-genetically induced mouse models of chronic inflammation would be useful to confirm the etiology of these changes. Another possible critique is that both genders of mice were not utilized for this study. Some prior metabolic studies of this mouse strain utilized male rather than female mice [22]. We chose female mice in part because evidence suggests that female mice may have lower variability in some biologic parameters than males [40, 41]. In addition, because frailty is more prevalent in older women than men in humans [42, 43], the use of a female mouse model is potentially more relevant to the aging-related conditions that are most pertinent to human frailty.

We acknowledge that this is a characterization paper rather than a mechanistic exploration. The exploration of the molecular mechanisms driving the phenotypic changes we see in adipose tissue and lipid metabolism will need to be more completely developed in future studies. The purpose of this study was to measure a battery of metabolic parameters in order to identify specific phenotypic characteristics that are altered across lifespan by chronic inflammation and aging in the IL 10^tm^ mouse. This in turn will inform future more mechanistic studies that may help to identify biological etiologies at the interface of chronic inflammation and aging and adverse health outcomes such as frailty, chronic disease, sarcopenia, and other conditions that shorten health span. In summary, the metabolic data presented in this manuscript helps to further characterize the older IL 10^tm^ mouse and provide rationale for its use as a model for the study of the impact of chronic inflammation and aging, frailty and late life decline.

## Supporting information

S1 Fig Oxygen consumption normalized by lean mass (top) and fat mass (bottom) in middle aged IL 10^tm^ (Panel A & B), young aged (Panel C & D). Each animal’s range of 48 hour VO_2_ is depicted by a single vertical group of points with a line connecting the means of each animal across each genotype.
(TIF)

S2 Fig Oxygen consumption normalized by total body weight (top) and lean mass (bottom) in old aged IL 10^tm^ (Panel A & Panel B). Each line connects the mean of each genotype (n = 10 per genotype).
(TIF)

S3 Fig Respiratory quotient in old IL 10^tm^ (Panel C), middle aged (Panel B) and young (Panel A). Lines connect means of each genotype (n = 10 per genotype).
(TIF)

S4 Fig Rearing (Z-Axis) activity measured during indirect calorimetry using an infrared beam grid & sensors. For young (Panel A), middle aged (Panel B), and old (Panel C).
(TIF)

S5 Fig Intra-peritoneal glucose tolerance test in young (Panel A), middle (Panel B) and old (Panel C) mice.
(TIF)

S6 Fig Oral glucose tolerance test in young (Panel A), middle (Panel B) and old (Panel C) mice.
(TIF)

S7 Fig Comprehensive plot of body weights of both groups across all ages. (TIF)

S8 Fig Metabolic panel. Plasma measurements of glucose (Panel A), and creatine kinase (Panel B).
(TIF)

S9 Fig Histogram of fat cell size in old IL 10^tm^ (Panel A) and control mice (Panel B).
(TIF)

S10 Fig Change in body weight and food consumed per gram bodyweight during indirect calorimetry for all groups. (TIF)

S1 Table Additional hematologic features of IL 10^tm^ and control mice. (DOCX)

S1 File CD11b macrophage/ monocyte immunohistochemistry protocol. (DOCX)
