# Supplementary figures and images for: Aged interleukin-10tm1Cgn chronically inflamed mice have substantially reduced fat mass, metabolic rate, and adipokines
Source: PLoS One. 2017 Dec 21;12(12):e0186811. doi: 10.1371/journal.pone.0186811 (PMC5739384; doi:10.1371/journal.pone.0186811)

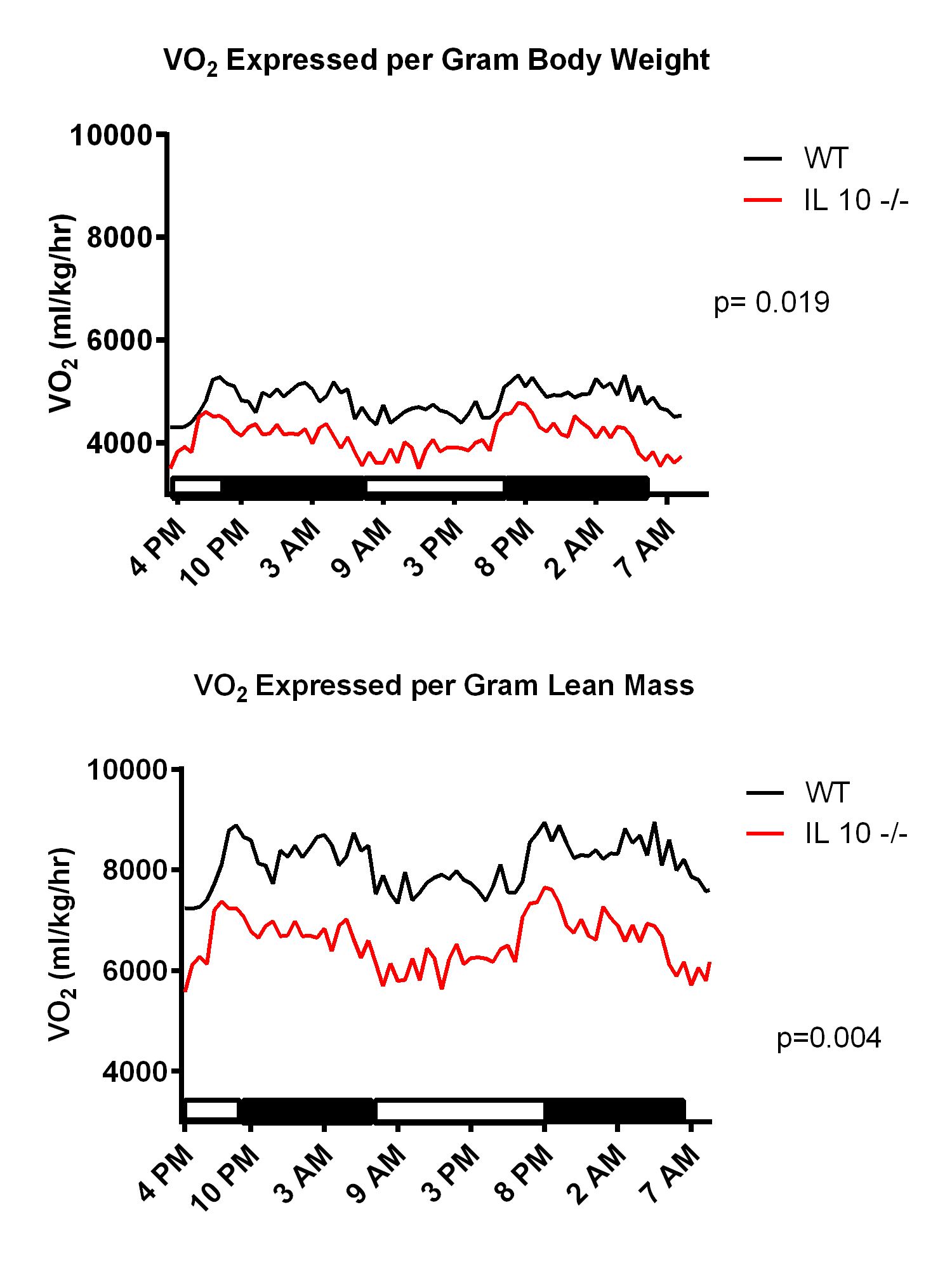

Supplement: S2 Fig — Oxygen consumption normalized by total body weight (top) and lean mass (bottom) in old aged IL 10tm (Panel A & Panel B). Each line connects the mean of each genotype (n = 10 per genotype). (TIF) [file pone.0186811.s002.tif]

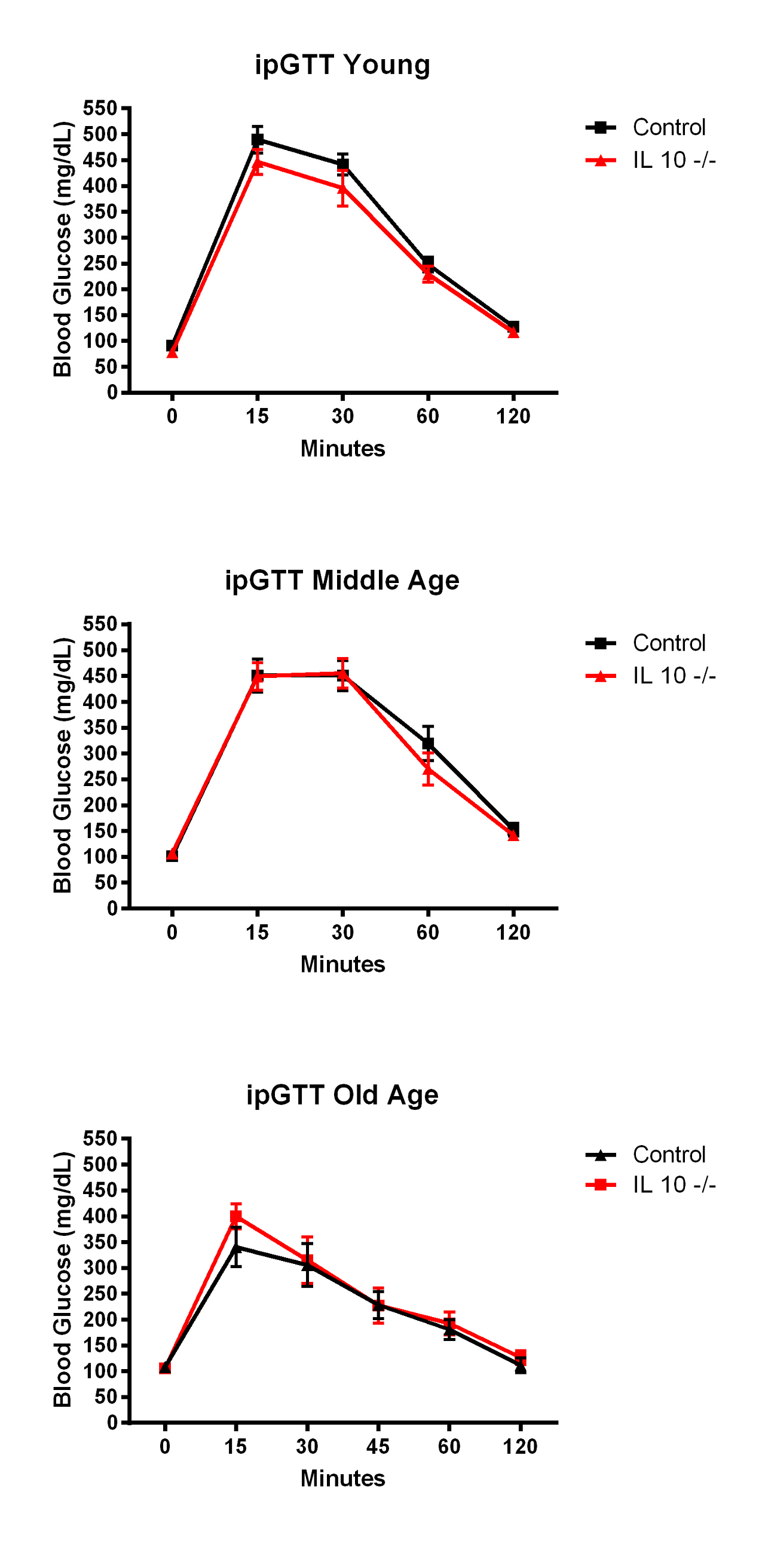

Supplement: S5 Fig — Intra-peritoneal glucose tolerance test in young (Panel A), middle (Panel B) and old (Panel C) mice. (TIF) [file pone.0186811.s005.tif]

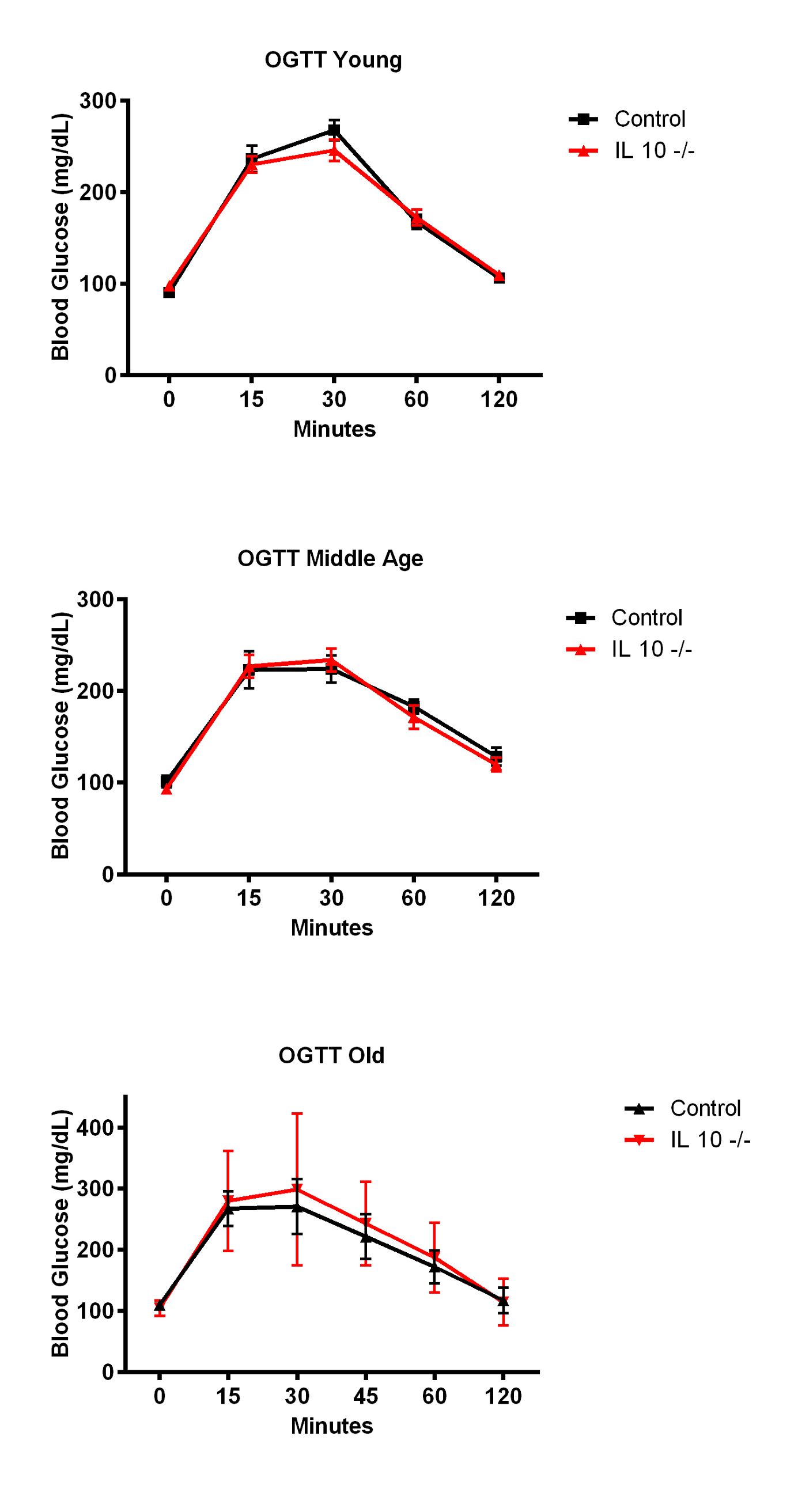

Supplement: S6 Fig — Oral glucose tolerance test in young (Panel A), middle (Panel B) and old (Panel C) mice. (TIF) [file pone.0186811.s006.tif]

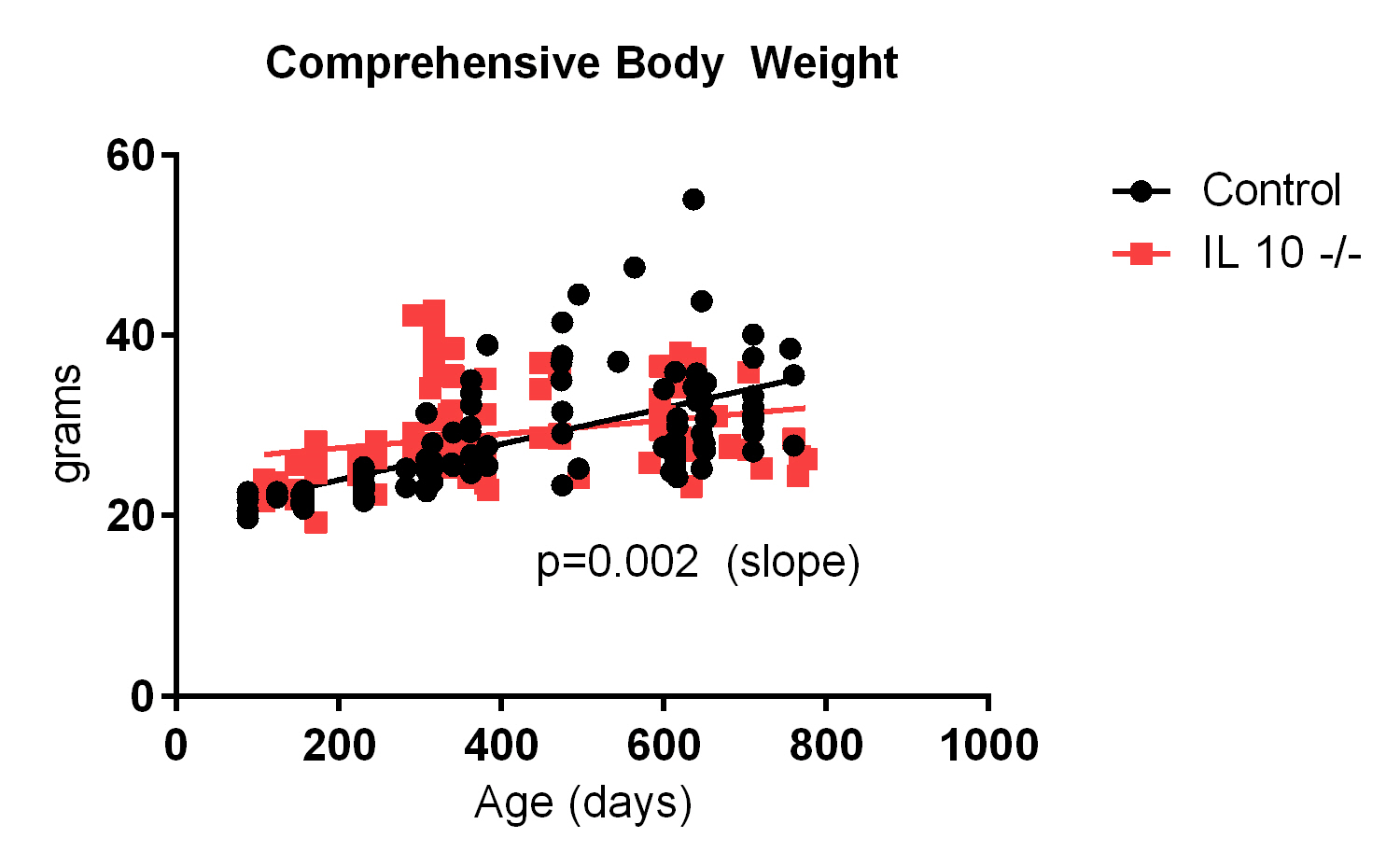

Supplement: S7 Fig — (TIF) [file pone.0186811.s007.tif]

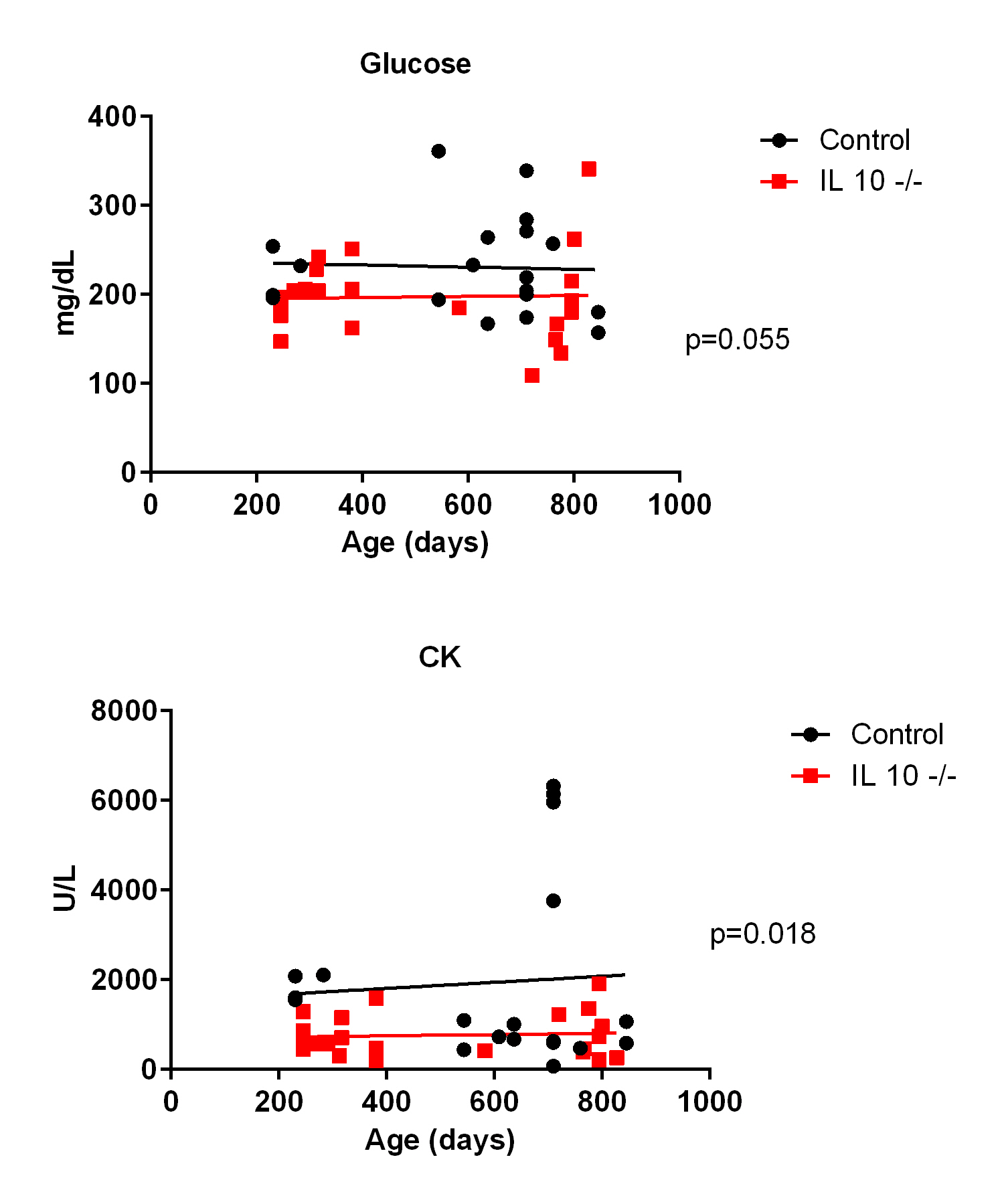

Supplement: S8 Fig — Metabolic panel. Plasma measurements of glucose (Panel A), and creatine kinase (Panel B). (TIF) [file pone.0186811.s008.tif]

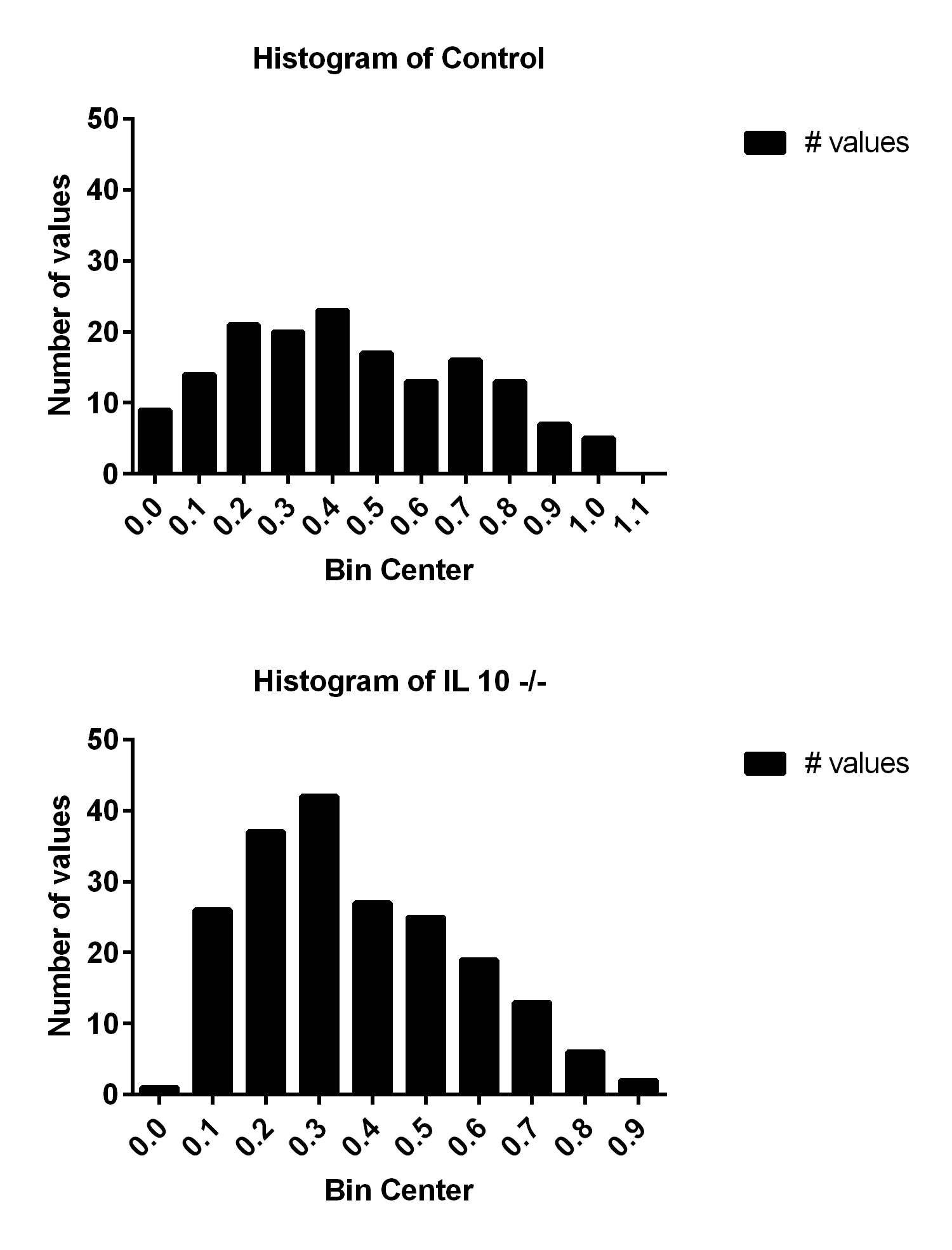

Supplement: S9 Fig — Histogram of fat cell size in old IL 10tm (Panel A) and control mice (Panel B). (TIF) [file pone.0186811.s009.tif]
