# Supplementary material for: Aged interleukin-10tm1Cgn chronically inflamed mice have substantially reduced fat mass, metabolic rate, and adipokines
Source: PLoS One. 2017 Dec 21;12(12):e0186811. doi: 10.1371/journal.pone.0186811 (PMC5739384; doi:10.1371/journal.pone.0186811)
Supplement: S1 Table — (DOCX) [file pone.0186811.s011.docx]

|  | IL 10 -/-  5 months  Mean  SEM  n=6 | C57Bl/6  5  months  Mean  SEM  n=6 | p-value | **IL 10 -/-**  **12 months**  **Mean**  **SEM**  **n=7** | **C57Bl/6**  **12 months**  **Mean**  **SEM**  **n=7** | **p-value** | **IL 10 -/-**  **22 months**  **Mean**  **SEM**  **n=6** | **C57Bl/6**  **22 months**  **Mean**  **SEM**  **n=6** | **p-value** |
| --- | --- | --- | --- | --- | --- | --- | --- | --- | --- |
| White Blood Cells | 4.72 ± 0.60 | 5.34 ± 0.99 | 0.60 | 4.8 ± 0.57 | 4.84 ± 0.61 | 0.98 | 5.81 ± 1.80 | 3.27 ± 0.39 | 0.20 |
| Neutrophil Number | 0.49 ± 0.06 | 0.58 ± 0.16 | 0.61 | 0.90 ± 0.27 | 0.81 ± 0.17 | 0.78 | 8.76 ± 0.48 | 8.55 ± 0.22 | 0.69 |
| Lymphocyte Number | 3.91 ± 0.53 | 4.39 ± 0.77 | 0.62 | 3.50 ± 0.34 | 3.58 ± 0.41 | 0.89 | 2.52 ± 0.64 | 2.29 ± 0.34 | 0.74 |
| Monocyte Number | 0.30 ± 0.04 | 0.31 ± 0.05 | 0.89 | 0.33 ± 0.04 | 0.30 ± 0.04 | 0.64 | 0.35 ± 0.14 | 0.30 ± 0.05 | 0.74 |
| Eosinophil Number | 0.0 3 ± 0.004 | 0.05 ± 0.03 | 0.24 | 0.07 ± 0.05 | 0.11 ± 0.06 | 0.64 | 0.06 ± 0.03 | 0.03 ± 0.01 | 0.34 |
| Basophil Number | N/A | 0.01 ± 0.01 | 0.34 | 0.02 ± 0.01 | 0.04 ± 0.02 | 0.53 | 0.02 ± 0.02 | 0.01 ± 0.01 | 0.74 |
| Eosinophil  (% of WBC) | 0.27 ± 0.06 | 0.69 ± 0.28 | 0.17 | 1.08 ± 0.62 | 1.91 ± 0.78 | 0.42 | 0.96 ± 0.41 | 1.13 ± 0.57 | 0.81 |
| Basophil  (% of WBC) | 0.04 ± 0.02 | 0.10 ± 0.05 | 0.32 | 0.30 ± 0.19 | 0.66 ± 0.26 | 0.29 | 0.30 ± 0.23 | 0.45 ± 0.32 | 0.71 |
| Hemoglobin | 13.35 ± 0.15 | 13.63 ± 0.17 | 0.25 | 13.44 ± 0.34 | 13.66 ± 0.32 | 0.67 | 11.40 ± 0.65 | 12.58 ± 0.34 | 0.15 |
| Hematocrit | 45.58 ± 0.29 | 45.53 ± 0.73 | 0.95 | 44.46 ± 1.29 | 44.23 ± 1.02 | 0.89 | 38.40 ± 2.40 | 39.50 ± 1.00 | 0.68 |
| Red Cell Distribution Width | 17.98 ± 0.29 | 17.40 ± 0.18 | 0.12 | 18.74 ± 0.24 | 18.30 ± 0.25 | 0.23 | 18.64 ± 0.48 | 18.76 ± 0.26 | 0.83 |
| Mean Platelet Volume | 4.27 ± 0.06 | 4.21 ± 0.04 | 0.51 | 4.65 ± 0.07 | 4.70 ± 0.09 | 0.72 | 4.920 ± 0.08 | 4.94 ± 0.12 | 0.89 |
