# Supplementary material for: Aged interleukin-10tm1Cgn chronically inflamed mice have substantially reduced fat mass, metabolic rate, and adipokines
Source: PLoS One. 2017 Dec 21;12(12):e0186811. doi: 10.1371/journal.pone.0186811 (PMC5739384; doi:10.1371/journal.pone.0186811)
Supplement: S1 File — (DOCX) [file pone.0186811.s012.docx]

*Supplemental Methods*

CD11b Immunohistochemistry

1. Deparaffinize and hydrate slides:

a. Preheat slides to melt paraffin if needed at 60C degrees for 10 min.

b. Transfer slides to staining rack and place them in xylene 2 X 10 min. each

c. Hydrate slides through graded alcohol – Absolute 2X, 95 2X, and one change of 70-dip until clear

d. Place slides in 1 change of dH2O then one change of dH2O with 0.1% Tween

e. Place slides in citrate buffer

f. Place slides into pap jar with Target Retrieval Solution – ready to use and steam for 45 min.

g. Let slides cool for 5 min.

h. Place slides into PBS with Tween (PBST)

2. Wipe off back of slides

3. Block with Dual Blocking solution – 5 minutes

4. Rinse with TBST

5. Apply CD11b (abcam; ab133357) primary – 1:10K for 45 min at Room Temp

6. Rinse with TBST

7. Apply Poly-HRP anti-Rabbit IgG – 30 minutes

8. Rinse with TBST

9. Apply DAB – 20 min (Sigma Fast DAB tablets one of each tablet and add 5ml dH2O and filter before use)

10. Rinse with TBST

11. Counter stain with Dako Mayers hematoxylin (1:5) – 1 minute

12. Running tap water wash – 5 min

13. Dehydrate, clear, mount and coverslip

a. dH2O, 70%, 2x 95%, 2x absolute, DUC, 2x xylene
